# Adapting and Operationalizing the RE-AIM Framework for Implementation Science in Environmental Health: Clean Fuel Cooking Programs in Low Resource Countries

**DOI:** 10.3389/fpubh.2019.00389

**Published:** 2019-12-20

**Authors:** Ashlinn K. Quinn, Gila Neta, Rachel Sturke, Christopher O. Olopade, Suzanne L. Pollard, Kenneth Sherr, Joshua P. Rosenthal

**Affiliations:** ^1^Fogarty International Center, U.S. National Institutes of Health, Bethesda, MD, United States; ^2^National Cancer Institute, U.S. National Institutes of Health, Bethesda, MD, United States; ^3^University of Chicago Medicine, University of Chicago, Chicago, IL, United States; ^4^Department of Medicine, Johns Hopkins University, Baltimore, MD, United States; ^5^Department of Global Health, University of Washington, Seattle, WA, United States

**Keywords:** RE-AIM, household air pollution, case studies, clean cooking, implementation science, program evaluation

## Abstract

**Introduction:** The use of models and frameworks to design and evaluate strategies to improve delivery of evidence-based interventions is a foundational element of implementation science. To date, however, evaluative implementation science frameworks such as *Reach, Effectiveness, Adoption, Implementation, Maintenance* (RE-AIM) have not been widely employed to examine environmental health interventions. We take advantage of a unique opportunity to utilize and iteratively adapt the RE-AIM framework to guide NIH-funded case studies of the implementation of clean cooking fuel programs in eleven low- and middle-income countries.

**Methods:** We used existing literature and expert consultation to translate and iteratively adapt the RE-AIM framework across several stages of the NIH Clean Cooking Implementation Science case study project. Checklists and templates to guide investigators were developed at each stage.

**Results:** The RE-AIM framework facilitated identification of important emerging issues across this set of case studies, in particular highlighting the fact that data associated with certain important outcomes related to health and welfare are chronically lacking in clean fuel programs. Monitoring of these outcomes should be prioritized in future implementation efforts. As RE-AIM was not originally designed to evaluate household energy interventions, employing the framework required adaptation. Specific adaptations include the broadening of *Effectiveness* to encompass indicators of success toward any stated programmatic goal, and expansion of *Adoption* to include household-level uptake of technology.

**Conclusions:** The RE-AIM implementation science framework proved to be a useful organizing schema for 11 case studies of clean fuel cooking programs, in particular highlighting areas requiring emphasis in future research and evaluation efforts. The iterative approach used here to adapt an implementation science framework to a specific programmatic goal may be of value to other multi-country program efforts, such as those led by international development agencies. The checklists and templates developed for this project are publicly available for others to use and/or further modify.

## Introduction

### The Health Potential of Cooking With Clean Fuels

Reducing the morbidity and mortality attributable to cooking with solid fuels (e.g., wood, dung, charcoal, and crop residues) and kerosene is a significant public health priority. Approximately 3 billion people currently cook with these polluting fuels, and exposure to household air pollution (HAP) from burning these materials is estimated to result in 2.6–4.3 million premature deaths a year (1, 2). Shifting to cleaner alternatives [e.g., liquefied petroleum gas (LPG), ethanol, biogas, and electricity] would result in progress toward multiple global goals, from improvement in public health to climate change mitigation (3). As the transition to cleaner cooking technologies has already occurred in higher-income countries, the existing imperative is therefore one of implementation: how do we achieve the extension of what is known (clean fuels reduce air pollution and protect health) to what is practiced (sustained and exclusive use of clean fuels for cooking), in the variety of settings where people rely on polluting solid fuels to meet their cooking needs?

There are numerous examples demonstrating the implementation gap that has impeded the achievement of health goals in the clean cooking sphere. For example, many programs have promoted “improved” stoves that still use relatively unprocessed biomass fuels such as wood and charcoal. While they may reduce fuel use, often can be produced locally, and may provide some reduction in air pollution, these stoves generally do not reduce pollution to the guideline levels established by the World Health Organization (WHO) that are understood to be required to minimize adverse health impacts (2). A shift in focus to stoves powered by “clean” fuels such as gas (biogas/LPG/natural gas), electricity, and in some cases processed biomass pellets (4) would help greatly in at least setting the stage for achieving the HAP reductions that are sought.

All these fuels require money to purchase, however, so financing for clean stoves and fuels is another area ripe for implementation research. As is true for many other development objectives, the populations most affected by HAP are often those least able to afford the financial investments required to transition to clean fuels (5, 6). Nonetheless, income has been shown to be less strongly associated with use of clean fuels than otherwise might be expected (7). Meanwhile, despite the fact that recent field and modeling studies show that exclusive or near exclusive use of clean fuels is required to achieve the WHO air quality targets (8, 9), adoption of clean fuels for cooking is often incomplete. Households regularly continue to cook with their traditional stoves even as they begin cooking with a new and cleaner stove (10), a practice called “stacking” which subverts the achievement of substantial air pollution reductions. Lastly, to achieve meaningful reductions in household air pollution, attention must also be paid to background ambient air quality that reflects larger, community-scale energy use and structural dynamics, and not just individual and household-level behaviors (11).

### The Clean Cooking Implementation Science Network's Case Study Project

The field of implementation science is well-suited to investigate these questions (12). Implementation science makes ample use of theories and frameworks, which have been shown to enhance the effectiveness of evidence-based health interventions (13) by informing development of nd implementation strategies that are adapted to different settings and improve intervention success (14, 15). Employing the tools of implementation science to better understand how to close the clean-fuel cooking implementation gap has been identified as a priority by the U.S. National Institutes of Health (16), which launched the Clean Cooking Implementation Science Network (ISN), https://www.fic.nih.gov/About/Staff/Policy-Planning-Evaluation/Pages/clean-cooking-implementation-science-network.aspx, in 2015 in partnership with the U.S. Agency for International Development (USAID), the Centers for Disease Control and Prevention (CDC), the Environmental Protection Agency (EPA), and the Clean Cooking Alliance (CCA). The network is composed of researchers working on issues related to household air pollution and cooking energy transitions hailing from a number of academic disciplines (e.g., environmental health, medicine, epidemiology, economics, anthropology, and ecology), as well as government officials from relevant agencies and ministries, representatives of clean fuel implementing organizations and NGOs, and experts in implementation science. The guiding aims for the network are to advance the science of uptake and scale-up of clean-fuel cooking technology in low-and middle-income countries and to foster collaborative efforts and understanding among researchers and implementers toward this end.

The Clean Cooking ISN's case study project was initiated after a series of meetings in 2016 with the ISN network and its Steering Committee. In these meetings, participants identified a notable lack of documented literature relating to specific cases of success and/or failure of clean-fuel cooking implementation efforts, despite the fact that clean fuel programs and clean cooking programs are rolling out around the world. The Clean Cooking Alliance, a network of partners invested in expanding adoption of clean cooking solutions, set an initial goal of fostering the adoption of clean cooking in 100 million homes globally by 2020 (17), a target that is likely to be exceeded. Meanwhile, efforts led by national governments and multinational organizations are promoting clean-fuel cooking solutions at a grand scale: India's Pradhan Mantri Ujjwala Yojana program, for example, reports that it has already expanded access to LPG to 85% of the national population (18). World Bank programs and other bilateral funders have also participated in funding and promoting clean-fuel cooking solutions. Despite all of this investment, however, evaluation of these programs has been minimal to date.

The ISN thus initiated a call for proposals in late 2016 for the development of case studies to evaluate clean fuel cooking programs in low and middle-income countries. Eleven programs were selected for development into case studies and were subsequently published as a Special Issue in *Energy for Sustainable Development*, titled “Scaling up clean fuel cooking programs in low and middle-income countries”(19). Briefly, the case studies comprise: four LPG scale-up initiatives, in Cameroon, Ghana, Indonesia, and Peru; two biogas programs, in Cambodia and East Africa; two compressed biomass projects, in Rwanda and China; two alcohol fuel programs, in Ethiopia and Nigeria; and a case study of energy transitions in Ecuador encompassing both a historical LPG effort and a more recent electric induction program (see Table 1).

**Table 1:** Clean fuel cooking program case studies.

**Case study title**	**Location**	**Cooking fuel**	**DOI**
Assessment of the Cambodian national biodigester program	Cambodia	Biogas	https://doi.org/10.1016/j.esd.2018.06.008
The Government-led initiative for LPG scale-up in Cameroon: programme development and initial evaluation	Cameroon	LPG	https://doi.org/10.1016/j.esd.2018.05.010
Development of renewable, densified biomass for household energy in China	China	Biomass pellets and briquettes	https://doi.org/10.1016/j.esd.2018.06.004
Government policy, clean fuel access, and persistent fuel stacking in Ecuador	Ecuador	LPG; electricity	https://doi.org/10.1016/j.esd.2018.05.009
A case study of the ethanol CleanCook stove intervention and potential scale-up in Ethiopia	Ethiopia	Ethanol	https://doi.org/10.1016/j.esd.2018.06.009
Ghana's rural liquefied petroleum gas program scale up: A case study	Ghana	LPG	https://doi.org/10.1016/j.esd.2018.06.010
The Mega conversion program from kerosene to LPG in Indonesia: lessons learned and recommendations for future clean cooking energy expansion	Indonesia	LPG	https://doi.org/10.1016/j.esd.2018.05.011
Africa biogas partnership program: a review of clean cooking implementation through market development in East Africa	Kenya, Tanzania, Uganda	Biogas	https://doi.org/10.1016/j.esd.2018.05.012
Building a consumer market for ethanol-methanol cooking fuel in Lagos, Nigeria	Nigeria	Ethanol/Methanol	https://doi.org/10.1016/j.esd.2018.06.007
An evaluation of the Fondo de Inclusión Social Energético program to promote access to liquefied petroleum gas in Peru	Peru	LPG	https://doi.org/10.1016/j.esd.2018.06.001
Implementation and scale-up of a biomass pellet and improved cookstove enterprise in Rwanda	Rwanda	Biomass pellets	https://doi.org/10.1016/j.esd.2018.06.005

### The RE-AIM Framework

We chose to organize the case study project around the commonly used implementation science framework, *Reach, Effectiveness, Adoption, Implementation, Maintenance* (RE-AIM) (20), in an effort to standardize data collection and reporting. RE-AIM is one of the most frequently applied implementation frameworks (21), and had previously been introduced to the ISN network at its initial network meeting in 2015. RE-AIM is often used to evaluate programs and thus was seen as appropriate to the largely retrospective nature of the case study project. Although RE-AIM has previously been used outside of health care systems [see (21, 22) for some examples], and the developers of RE-AIM have been actively engaged in exploring applications of the framework in a diversity of settings (23), applications of RE-AIM in low- and middle-income countries (LMICs) are still relatively uncommon. To date there are also relatively few examples of the use of RE-AIM in the field of environmental health [see (24)]. The ISN felt that using RE-AIM to guide the case study project was an opportunity not only to learn generalizable lessons about clean cooking programs and compare case studies across countries, but also to provide the field with information that would advance the use of RE-AIM in LMIC settings.

The RE-AIM framework posits that public health impact of an evidence-based intervention will be achieved if an EFFECTIVE intervention REACHes a broad and representative segment of the population by being ADOPTED by willing organizations and staff, IMPLEMENTED as intended, and MAINTAINED over time by organizations and individuals. Each of the five elements, thus, is equally important to success as measured by public health impact—and importantly, data associated with all five aspects are essential to understanding the success, or failure of any implementation effort and to generalize from this experience to other settings. Initially used primarily as an evaluation tool for health behavior research, RE-AIM has expanded to cover diverse public health content and multiple research stages, including planning and study design, as well as assessment and evaluation of programs and policies (22, 25). Here, we discuss how we used RE-AIM to develop a generalizable framework for use in the evaluation of clean fuel adoption programs in LMIC settings.

## Methods

**Figure 1:**
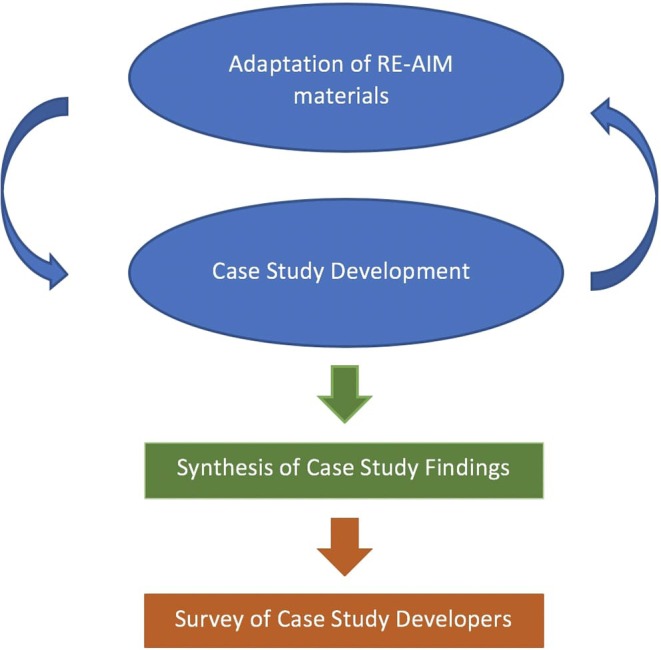
Flow chart of activities.

RE-AIM was used at each stage of the case study project, namely: during the call for proposals, proposal evaluation and selection, data collection, manuscript writing, and summarization. The framework for clean-fuel cooking was iteratively adapted as the project progressed (see Figure 1). The main outputs of this process were two RE-AIM templates: first, a checklist used during the proposal stage (see Table 2); and second, a data collection template to guide case study teams in gathering and summarizing data for each RE-AIM dimension (see Table 3). The checklist in Table 2 contained fields for case study developers to indicate the availability of data pertaining to each RE-AIM dimension, indicating whether the data were qualitative or quantitative in nature and a description of plans to collect any data that were not pre-existing.

**Table 2:** Initial RE-AIM checklist developed for case study proposals.

**Dimensions/Data elements**	**Available?** **Quantitative or Qualitative**
**REACH (scale and coverage of intervention)**	
Description of target population (geographic coverage, numbers targeted, demographic characteristics)	
Duration/dates of intervention project/programme	
Setting characteristics (urban vs. rural, seasonal climate, access to roads, and transport infrastructure, etc.)	
Percent individuals/households reached based on target population	
Characteristics of households reached compared to non-participants or to target population (e.g., baseline fuel/s used, socioeconomic characteristics, education etc.)	
Other factors that affect reach of program including policy context, program budget constraints, conflict, fuel availability, and cost.	
**EFFECTIVENESS (ability of fuel/technology to achieve desired goals)**	
Description of clean cooking intervention fuel/technology (relate to IWA's Tiers and/or ISO standards if possible)	
If available, from literature or measured in the field (please address availability of each item):Measures of stove emissions Measures of household/personal air pollution exposure before and after intervention Measures of safety (e.g., burns) before and after intervention Measures of fuel and/or time savings Measures of impact of the intervention on desired health outcomes	
**ADOPTION—Program and Societal level (factors influencing adoption of the clean cooking intervention)**	
Description of financial, tax, and subsidy aspects and how these have affected adoption and use over time (including cost of intervention to end-users and price comparison for other available energy alternatives)	
Description of supply chain (from fuel/stove production to fuel/stove distribution, consistency of supply etc.), and how these have affected adoption and sustained use	
Description of market development (e.g., promotional strategies, aspects influencing business expansion), and how these have affected adoption and sustained use	
Description of regulation and legislation (particularly around fuel supply, distribution and enforcements effectiveness of market rules), and how these have affected adoption and sustained use	
Description of policies, programmatic and policy mechanisms, and how these have affected program implementation and adoption	
Other factors important to adoption at the program and societal level	
**ADOPTION – Household and Community level (factors influencing adoption of the clean cooking intervention)**	
Measure of household use of technology, including if possible, degree of fuel, or stove stacking	
Perception of affordability, Willingness To Pay measures	
Perceived benefits and/or disadvantages of the intervention, and influence of these perceptions on adoption and sustained use. Important aspects to consider are perceptions of the intervention's effect on: health cooking time opportunity cost cleanliness safety quality of food prepared other	
Accessibility/reliability of fuel supply, and its effect on adoption and sustained use	
Other factors important to adoption at the household and community level	
**IMPLEMENTATION (How the program is rolled out and scaled up)**	
Description of implementation strategy including underlying theory, if any, and how it may be integrated with any other interventions (e.g., sanitation, antenatal services)	
Implementing agency / organization / company etc. (or a combination of these)	
Cost of intervention (time or money) from the implementer perspective	
Consistency of implementation across staff/time/settings/subgroups (not about differential outcomes, but process)	
Preparation for reliability of supply chain and price fluctuations	
Community involvement; including women's engagement, and how these factors have affected adoption and sustained use of the intervention	
User and/or provider training	
Adaptations made to intervention during program/project roll out (i.e., was the intervention delivered as intended?)	
Other factors important to implementation, including policy and regulatory environment.	
**MAINTENANCE—Household and community Level (how well the intervention is sustained at the household/community level)**	
Indicate availability of data for each category and the time frame for initial and follow-up data (Ideally at 6 months to a year after initial intervention): Measure of air pollution exposure (with or w/o comparison to a public health goal) and follow-up after final intervention contact Measure of stove use (with or w/o comparison to a benchmark) Measure of fuel use (with or w/o comparison to prior) Measure of attrition (%) and differential rates by demographic/geographic characteristics or treatment condition Measure of stove breakdown/repair Measure of continued financial investment in the intervention by the household or community	
Other factors important to maintenance at the household and community	
**MAINTENANCE—Program and societal Level (factors influencing the sustainability of the intervention at the program level)**	
Availability/ accessibility of intervention over time, and importance of these factors to adoption and sustained use	
If program is still ongoing at ≥12 months post intervention funding (provide timeframe)	
If and how program was adapted subsequently (which elements retained AFTER program completed)	
Some measure/discussion of alignment to organization mission or sustainability of business model	
Description of long-term repair and maintenance infrastructure, including forms of post-acquisition support, and their effects on adoption and sustained use)	
Description of any long-term subsidies/incentives and plans for continuity or phase-out, and their effects on adoption/sustained use	
Other factors important to maintenance at the program and societal level	

**Table 3:** Simplified RE-AIM data gathering template for clean cooking programs.

**RE-AIM dimension**	**Definition**	**Case study-specific metrics**
Reach	No. of people and percentage of the target population affected. The extent to which the individuals reached are representative and include those most at risk.	Absolute numbers and characteristics of the target population Number of people/households and percentage of the target population that have been reached. How do the characteristics of the people reached differ from target population? How do the characteristics of the people reached differ from target population? Duration/dates of the program Sociodemographic trends that affect program (e.g., migration etc.)
Effectiveness	A measure of effects, including positive, negative, and unanticipated consequences.	Toward program goals Stated goals of the program Success achieved toward each of the stated goals Unanticipated consequences Toward health improvements POTENTIAL of the program for achieving improvements in health (e.g., ISO tier of the technology; exposure reductions; baseline levels of HAP related diseases; etc.) Degree to which technology displaced polluting fuels in target populations c. If health data available, were there changes associated with program
Adoption (inclusion and approval)	No. and percentage of settings participating, and the extent to which the settings selected are representative of settings that the target population will access.	How were program sites selected? Who was involved in selecting implementation sites and was this an inclusive process? Were the implementation agents viewed positively or negatively by the communities? How much fuel stacking in those homes that did take up new technology Perceptions of affordability, perception of intervention benefits, and/or disadvantages
Implementation	Level of adherence to implementation principles or guidelines, the extent to which all vs. selected elements are implemented, and the cost.	Policy context Who financed, and who implemented? Monitoring process and measures Cost of the program, over what time period? (To the program leadership. Could be total cost, cost per capita, or cost projection) Major changes to program targets/goals/drivers/timelines that occurred during implementation, and why did they occur?
Maintenance	Individuals continue to exhibit the desired behavior changes; change is maintained; development of new barriers to use is prevented or mitigated.	To what extent has the reach of the program been maintained over time? (e.g., households still using the technology at least 1 year post-adoption vs. abandoning it). Ongoing access to fuels? Supply side and cost to consumer. Indicators of program's sustainability? Risks to sustainability?

### Development of Templates for Case Study Proposal Selection and Case Study Development

The development of each of the two templates (the RE-AIM checklist and the data collection template) occurred iteratively. A case study working group comprised of ISN leadership and interested ISN members convened in a series of virtual meetings and via email correspondence to develop and refine these templates. The working group members were academics and government officials trained in a variety of specialties spanning the health sciences (epidemiology, environmental health, medicine, global health), social sciences (economics, anthropology, and management), and implementation science. In developing the templates, we consulted existing RE-AIM material (e.g., that available on the website re-aim.org) and prior literature on the use of RE-AIM in environmental health and community-based applications [e.g., (24, 26)]. We used these pre-existing materials alongside our prior knowledge of clean cooking programs to generate indicators that were thought to be relevant to the case study project.

Over time, we iteratively modified the indicators based on feedback and the experiences of the case study teams. For example, each prospective case study team submitted a RE-AIM checklist (Table 2) along with their case study proposal. When the working group reviewed these checklists, we noted areas of potential overlap, points of confusion, and categories that were commonly reported as “data not available” across the proposals. We then used these learnings to create the case study development template (Table 3). Lastly, we made small modifications to each template prior to presenting them in this manuscript to further refine and clarify any elements that had presented any confusion during the development of the case studies. Elements that contributed to these iterative changes included the availability of data, clarity of indicators (and differentiation from other indicators), and qualitative and quantitative feedback from the case study authors.

### Synthesis of Case Study Findings

After the case studies had been developed, ISN leadership consolidated and edited the 11 RE-AIM data tables submitted alongside the narrative case studies into a single summary spreadsheet that was published with the Special Issue in *Energy for Sustainable Development* (19).

### Perceptions of Case Study Developers

We gathered the perceptions of the case study developers on the utility of RE-AIM for the case study project using a questionnaire. The questionnaire consisted of 13 questions covering the following general areas: (1) prior experience with RE-AIM; (2) Perceived ease and usefulness of employing RE-AIM for this project; (3) Challenges presented by the particular RE-AIM constructs (reach, effectiveness, adoption, implementation, maintenance); (4) Impact on future work. The questionnaire employed a mixture of question types, including multiple choice, Likert scale, ranking, and open-ended responses and was deployed to the case study developers using an online survey platform. The full set of survey questions can be found in the Supplemental Material. Eighteen case study developers provided feedback using the online questionnaire, and this feedback was synthesized and analyzed by the authors of this manuscript. Analysis of responses consisted of summary statistics (for quantitative items) and grouping of responses by theme and content (for qualitative items).

The clean cooking fuel case studies that employed the adapted RE-AIM tool were reviewed and approved through the institutional review boards (IRBs) of their respective lead investigators. Feedback from the case study investigators regarding the utility of this tool was treated as exempt, and the use of this data in this manuscript was cleared by the Fogarty International Center at the U.S. National Institutes of Health.

## Results

### Adapted RE-AIM Templates

Outputs of this project include the RE-AIM checklist (Table 2) and data collection template (Table 3) created for the case study developers. In the initial checklist (Table 2), general RE-AIM indicators were combined with domain-specific information about clean fuel cooking programs and policies. For example, the checklist asked for ratings of the stove and fuels used according to the International Organization for Standardization's Interim Workshop Agreement Guidelines for evaluating cookstove performance (27). We also asked for information about fuel supply policies, stove stacking, and women's engagement in implementation efforts. Some of these indicators were drawn from a framework of Adoption Indicators previously generated by the Clean Cooking Alliance (28).

Table 3 is the RE-AIM data collection template that was provided to case study developers to define case study metrics across the five RE-AIM dimensions as part of case study development. This template was informed by the information collected at the proposal stage (in the submitted Table 2 checklists). In some cases, alternative metrics were generated for data that were indicated in Table 2 as unlikely to be available. For example, the submitted Table 2 checklists indicated that health outcomes data were very seldom available. Due to this lack of data availability, the corresponding metric in Table 3 became one related to the *potential* health impact of the stove/fuel combination that was utilized in the case (relying, for example, on laboratory, and field emissions testing data conducted elsewhere for the same stove/fuel combinations being deployed; these data are often used to estimate health benefits that would be expected to accrue from reductions in exposure to particulate matter and other compounds). A comprehensive table of the RE-AIM data gathered across the eleven case studies can be found in Quinn et al. (19).

### Synthesis of Case Study Findings

**Figure 2:**
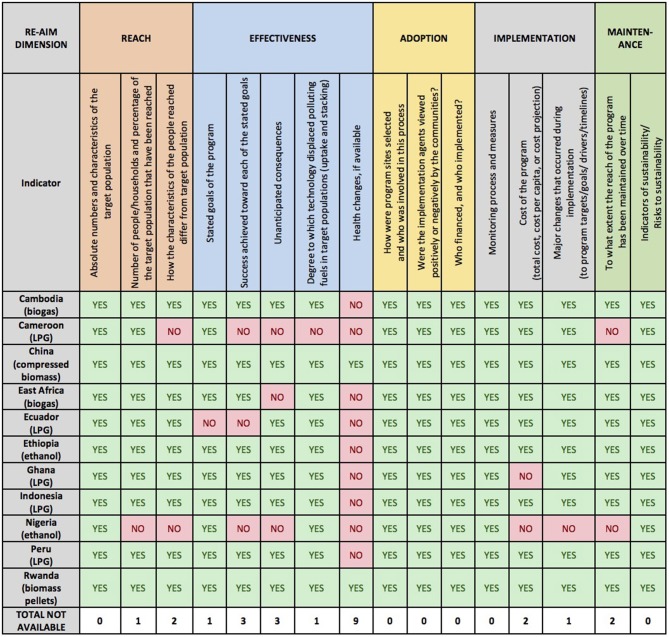
Summary of RE-AIM data availability across 11 case studies.

Figure 2 presents a summary of the availability of data for each RE-AIM dimension. In general, data were widely available for all five RE-AIM dimensions. Data to address *Adoption* (defined for the purposes of this project) was the most widely available, with no case studies reporting a lack of access to data related to this RE-AIM dimension. Data pertaining to *Reach, Implementation*, and *Maintenance* were also widely available. Across the 11 case studies, data was least available for the *Effectiveness* dimension. This was especially true for data concerning health outcomes—only two of the 11 case studies were able to report data on health impact, and these were on a limited scale. Nine of the case studies were not able to gather any data related to health outcomes. Other aspects of *effectiveness* that related to programmatic goals were sometimes unavailable as well, with three case studies each reporting a lack of data related to “success achieved toward each of the stated goals” and “unanticipated consequences.” The prospective nature of several case studies, e.g., Cameroon (29) and Nigeria (30), meant that less data were available across all dimensions to track RE-AIM indicators for these cases in particular.

### Perceptions of Case Study Developers

Perceptions of case study developers on using RE-AIM for this project were assessed using an online questionnaire. A total of 18 case study developers, including representatives from all 11 case studies, contributed their feedback on the utility of the tool. Despite the fact that the RE-AIM framework had been introduced to the ISN at a meeting in 2015, a number of case study developers did not attend that initial meeting. Thus, of the 18 respondents, the majority (12, or 67%) had never heard of RE-AIM prior to the case study project. Four respondents had heard of RE-AIM but had never used it, and only two had used it in a previous project. Nonetheless, 9 respondents (50%) found it “easy” to use, while seven found it “neither difficult nor easy,” and only two found it “difficult,” or “very difficult.”

**Figure 3:**
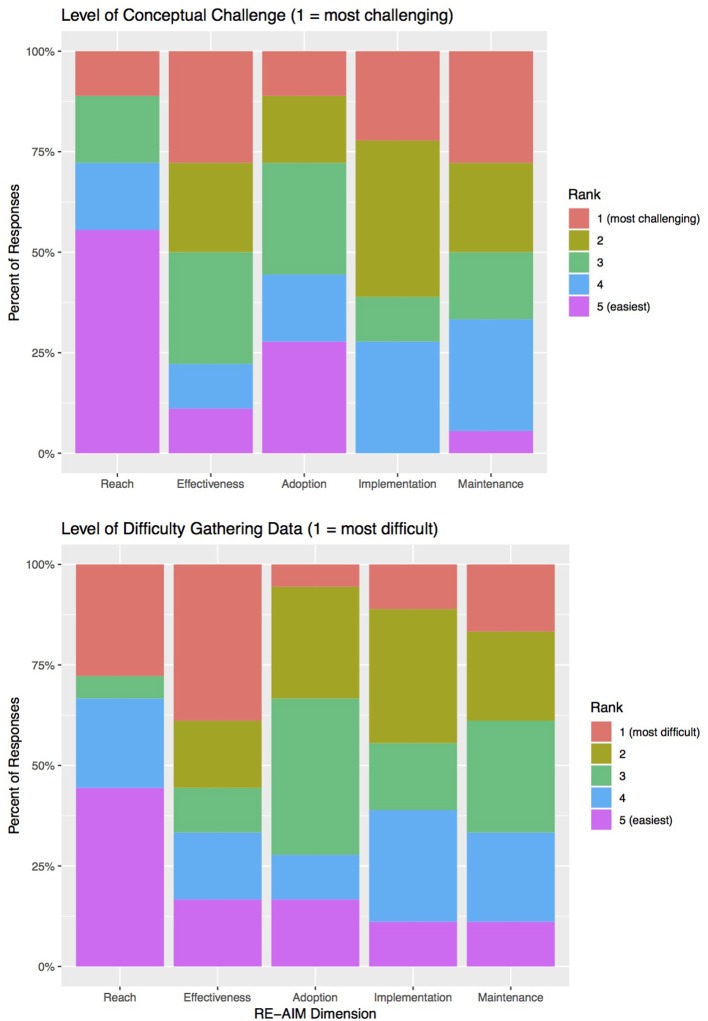
Case Study Developers' surveyed responses to questions about the conceptual challenge of, and difficulty gathering data for, the five RE-AIM dimensions. *N* = 18 responses.

Figure 3 shows how the case study developers ranked the different RE-AIM dimensions according to two factors: (a) level of conceptual challenge to understanding the dimension as it applied to their case; and (b) difficulty in gathering data for their case study. Case Study developers consistently ranked *Reach* as the least challenging dimension both for applicability to the case and for the ease of gathering relevant data. They found *Effectiveness, Implementation* and *Maintenance* to be the most challenging both to apply to the case and in terms of difficulty collecting relevant data for each dimension. This was because certain case studies were of programs at a nascent stage (with little implementation, maintenance, or outcome data yet available), and/or because of a perceived lack of fit between RE-AIM's emphasis on “program” implementation and the national-level policies and regulations that drove some cases. Across both the prospective and retrospective case studies, it was most difficult to gather data for *Effectiveness* (with 10 out of 18 respondents, or 55%, ranking the dimension as among the top two “most difficult” dimensions in terms of gathering data).

Qualitative responses to open-ended questions in the survey enhance understanding of the reported challenges. For example, the reported challenges in understanding how to incorporate the dimensions of *Implementation* and *Maintenance* appear to derive from the fact that the case study project included several evaluations of clean-fuel cooking programs that had not yet been fully implemented, making evaluation of these facets difficult. Comments along these lines included, for example:

- *The work on the ground is still in progress, so we were not yet able to report on many of the metrics*.
- *It seemed challenging to provide responses within the framework for programs that are just getting started and are anticipated to be ongoing and changing, rather than at steady state*.

Difficulty in gathering data related to effectiveness often related to the fact, as discussed above, that these clean-fuel cooking programs were uniformly launched with goals that did not place health improvement at the forefront. They differ, therefore, from more clinically or public-health-oriented programs where effectiveness—in terms of achievement of health improvement—is easier to assess. A number of comments spoke to this, for example:

- *My feeling was the RE-AIM was designed for a more clinical outcome and did not completely fit the context for household energy*.
- *RE-AIM assumes that the program driver is health, but of course often in cookstoves health is a co-benefit rather than the primary outcome*.

Some case study developers additionally felt that it was difficult to fit certain contextual and implementation factors within the RE-AIM framework that were key to the case. Aspects of the case studies that the authors felt were difficult to fit in to RE-AIM included:

- The political and socio-cultural circumstances that circumscribe the subsidies
- *The presence of a charismatic, committed leader*.
- *Use of behavior change concepts and techniques*.
- The strategy for creating the conditions for investment
- ‘*Logistical’ issues with clean cooking” (supply side of the fuel)*
- Driving factors for decisions that were made politically
- *Specific barriers to adoption and factors that can drive a wedge between adoption and health-relevant exposure reduction*.

Despite these challenges and their relative lack of experience with RE-AIM, case study developers reported that the framework was useful to various aspects of the case study development process, as shown in Table 4. Using RE-AIM for understanding data availability was the aspect most commonly reported as being “very” or “extremely” useful (reported by 67% of respondents), followed by planning for data collection (56%). In their qualitative comments, however, case study team members also reported that RE-AIM was useful for comparisons across case studies:

- *I do see that having a common framework among the case studies is quite beneficial*.
- *I found the RE-AIM summaries helped greatly in structuring information about quite complex and very different projects—a real asset*.
- *It has been a very useful tool for comparing across case studies*.

**Table 4:** Reported usefulness of RE-AIM for different aspects of the case study project.

		**Not at all *n* (%)**	**Somewhat/** **Moderately *n* (%)**	**Very/** **Extremely *n* (%)**
How useful did you find RE-AIM for:	Understanding data availability	1 (6)	5 (28)	12 (67)
	Planning for data collection	1 (6)	7 (39)	10 (56)
	Understanding factors that led to the success or failure of the case	2 (11)	9 (50)	7 (39)
	Drawing generalizable conclusions that extend beyond the case	1 (6)	9 (50)	8 (44)
	Structuring the manuscript	2 (11)	10 (56)	6 (33)

Lastly, case study developers were asked to evaluate whether the experience of using RE-AIM for the clean fuel cooking case study project would lead them to approach their work differently in the future. Here, 13 out of 18 respondents (72%) replied “Yes,” with some of the specific ways that RE-AIM would influence future work outlined below:

- *REAIM could help us broaden our view a little and possibly adjust some of our study design to be a bit more holistic*.
- *Taking a more holistic approach to data collection*.
- *More emphasis on measures for sustained use*.
- *I appreciated the variables/indicators identified under each heading, and this helped organize my thoughts*.
- *Having a suitable structured framework that covers all aspects of the initiative is very valuable, both for the specific example, but also for making comparisons with others*.

## Discussion

Clean-fuel cookstove programs are being rolled out on a massive scale, and a consolidated method for evaluating these initiatives is needed both for individual programs and also to enable cross-initiative comparisons. The NIH clean fuel case study project showed us that the RE-AIM framework has utility for these purposes, particularly with the adaptations that were made here.

The need to adapt RE-AIM for this project was not unlike previous efforts to employ RE-AIM for environmental health interventions. For example, King et al. (24) note that many of the RE-AIM dimensions are difficult to apply to environmental health interventions, such as those meant to affect air quality or improvements to public space. For example, how to calculate the “Reach” of an intervention that improves sidewalks? How to define the settings at which “Adoption” occurs in the context of an intervention targeting outdoor air pollution? Similar challenges—such as defining reach and measuring compliance—have been discussed when it comes to the use of RE-AIM for policy applications, which have some overlap with the case studies here. In the case of policy applications, enforcement is an important aspect of implementation that can directly affect compliance and strongly influence success [see (25) for examples]. In this set of clean fuel case studies, certain initiatives, such as Indonesia's “zero kero” plan, benefited from policy-like structures and robust enforcement measures, while other programs relied more on ground-up marketing and diffusion approaches that did not have the benefit of strong enforcement measures to enhance compliance and implementation.

Notable dimensions of the RE-AIM framework that required adaptation for use in the clean fuel case study project included *Effectiveness* and *Adoption*. First, translating *Effectiveness* for this project required acknowledgment that clean fuel scale-up initiatives have largely been driven by goals outside the health domain, e.g., pertaining to the environment and economic concerns. For example, Indonesia's “Zero Kero” program was designed to phase out highly-subsidized kerosene and thus provide savings to the national budget (31), while the aims of Ghana's rural LPG program included reducing deforestation, reducing drudgery, and creating jobs, as well as reducing the health impacts of cooking with wood and charcoal (32). We therefore proposed case study metrics for this dimension that covered effectiveness in two areas: not only effectiveness related to the reduction of household air pollution and associated health improvement, but also effectiveness in relation to the goals as put forth by the specific clean-fuel cooking program (however those may have been stated).

*Adoption*, in the context of clean-fuel cooking, presents a different problem since the term “adoption” is widely used in this field to refer to individual-level initial uptake of a new cooking technology, e.g., (2, 16, 33–35). This conflicts with the RE-AIM definition of adoption situated at the organizational and setting level. Defining the “setting” of a clean-fuel cooking program presented its own challenges as many programs are not managed by a clear intermediary organization (as would be the case, for example, in an intervention operating through a hospital or clinic to meet patient needs). Rather, many clean-fuel cooking programs are defined by geography or demographics (e.g., income). For the *Adoption* dimension of RE-AIM we therefore chose to focus on “inclusion and approval,” as suggested in King et al. (24). We developed metrics here that focused on how the program rollout was determined, who was involved in these decisions, and how the implementing agents were viewed by the community.

To minimize confusion for the clean cooking community who use adoption to mean household-level uptake of technology, we also included metrics within *Adoption* that pertained to cooking technology usage at the household level. An important aspect of clean fuel adoption in terms of achieving health gains is the distinction between uptake (adding a stove) and displacement (replacing a stove). This has important implications: without discontinuation of the use of polluting fuels for cooking, exposure to health-damaging emissions may not be sufficiently reduced to improve health outcomes [e.g., see (8)]. In the clean cooking research community the practice of using multiple types of stoves within a household (adding new stove technology to an existing mix, rather than replacing the older cooking technology with the newer one) is termed “stacking.” In the RE-AIM framework, the practice of stacking fuels could theoretically fit either into adoption (where it pertains to initial decisions upon adoption of a new technology) or implementation (where it pertains to patterns of use over time). The decision to include these activities in “adoption” in this project are justified by the fact that we considered fuel choice and fuel usage—including decisions to stack fuels—as intrinsic to the potential adoption process and not merely as patterns that emerge over time. Initial adoption is often only partial adoption. We also asked about household-level perceptions of the new cooking technology as part of *Adoption*, since these perceptions are important determinants of uptake and use of new cooking technology (36).

The remaining RE-AIM dimensions were less in need of adaptation for this purpose, although we included a metric in *Maintenance* focused on fuel supply (covering ongoing access to fuels and the cost to the consumer over time).

Despite its comprehensiveness, case study developers identified a number of aspects crucial to understanding their cases that were difficult to fit in to the RE-AIM framework, even after adaptations of data collection tools and templates to fit the household energy context. Some of these missing factors had to do with the larger sociopolitical context in which the cases were embedded. Notable missing elements included: “The political and socio-cultural circumstances that circumscribe the subsidies,” “driving factors for decisions that were made politically,” and the impact of “the presence of a charismatic, committed leader.” “Logistical” issues (e.g., all the steps involved in distributing clean fuels to customers and ensuring steady supply) were also mentioned as hard to fit into the RE-AIM framework, along with specific barriers impeding the transition to clean fuels for cooking, and the potential role of behavior change interventions in overcoming these barriers.

The fact that aspects of the contextual setting that are essential to implementation success were difficult to capture in RE-AIM has been noted by other researchers, and in fact RE-AIM extensions such as PRISM (37) combine RE-AIM outcome measures with other dimensions crucial to success, including “external environment” and “implementation and sustainability infrastructure.” In future applications of RE-AIM to complex community-based programs, we might suggest that researchers and program evaluators consider using PRISM or another RE-AIM extension to more comprehensively evaluate those aspects of the contextual environment that are difficult to describe using RE-AIM alone.

Using RE-AIM for the case study project also highlighted the fact that some key outcome data—in this case particularly pertaining to long-term program maintenance and health outcomes—was not routinely monitored and thus unavailable. This data gap highlights the need for engaging the health sector in longitudinal monitoring and evaluation of clean-fuel cooking initiatives. Current programmatic evaluation might focus, for example, on the number of stoves distributed. Such simplistic metrics, however, do not come close to covering the complexity of the processes related to adoption and sustained use of clean fuel cooking technologies. For example, in addition to tabulating the initial distribution of a clean-fuel cooking solution, it is imperative to also investigate whether households use the stoves, whether they continue to use them over time, and whether the use of the stoves is exclusive or in conjunction with other, polluting stoves and fuels. Employing systematic approaches, ideally with common metrics, will greatly enhance the ability of the international development community to evaluate projects taking place around the world against national, bilateral and global targets, for example targets associated with the Sustainable Development Goals (38).

The overall approach of this case study project was to engage interdisciplinary teams of researchers who employed RE-AIM in a complementary fashion with additional tools to enhance the value of the project by providing data on these additional dimensions of context and climate. This approach could certainly be extended to additional domains beyond clean fuels for cooking. Meanwhile, the specific adaptations and templates developed for this project could be useful starting points to guide future researchers in the household energy domain who are interested in program planning and/or evaluation.

## Conclusions

Implementation science frameworks such as *Reach, Effectiveness, Adoption, Implementation, Maintenance* (RE-AIM) have been shown to enhance the effectiveness of interventions and can be used to evaluate factors associated with implementation success. This is the first known example using RE-AIM to evaluate clean fuel cooking programs in low- and middle-income countries.

Utilizing RE-AIM for the clean cooking community required adapting and operationalizing the framework. Specific adaptations included: specifying the metrics that would be able to inform each of the RE-AIM dimensions, taking account of the pre-existing meaning of some terms (e.g., adoption) in the clean cooking community, and broadening certain dimensions (e.g., effectiveness) to capture program-relevant outcomes. Case study developers found RE-AIM to be useful and relatively easy to use for gathering data and evaluating the clean fuel initiatives. The case study teams reported particular value from the RE-AIM framework when it came to comparing common elements of disparate programs. In the future, RE-AIM extensions such as PRISM might be useful to consider when evaluating community-based interventions to capture aspects of the contextual environment that were difficult to describe using RE-AIM alone.

Key findings from the case study project suggest that long-term monitoring and evaluation of clean-fuel cooking scale-up programs is often lacking, particularly regarding indicators relevant to sustained use of new cooking technology. Health outcome measures and measures of air pollution reduction are also insufficiently tracked. A recommendation to future implementers and evaluators of clean fuel cooking programs is to build infrastructure into their programs that will ensure middle- and long-term monitoring of these key indicators of implementation success.

Finally, this effort demonstrates how a commonly used implementation science framework can be adapted for use in low-and middle-income settings and in contexts where programs are not specifically driven by health objectives. Employing frameworks like these can yield robust program evaluations that can be used to assess program performance in light of national and international goals.

## Data Availability Statement

All datasets generated for this study are included in the article/Supplementary Material.

## Author Contributions

AQ and JR managed the project. AQ, JR, GN, and RS contributed to the design of the RE-AIM templates. AQ and GN compiled the data. AQ drafted the manuscript. CO, SP, and KS contributed sections of the manuscript. All authors contributed to manuscript revision, read and approved the submitted version.

### Conflict of Interest

The authors declare that the research was conducted in the absence of any commercial or financial relationships that could be construed as a potential conflict of interest.
