# Supplementary material for: Adapting and Operationalizing the RE-AIM Framework for Implementation Science in Environmental Health: Clean Fuel Cooking Programs in Low Resource Countries
Source: Front Public Health. 2019 Dec 20;7:389. doi: 10.3389/fpubh.2019.00389 (PMC6932973; doi:10.3389/fpubh.2019.00389)
Supplement: Supplementary file 1 [file Table_1.DOCX]

**Case Study Developer Survey Questions**

|  | **Question/question group** | **Response choices** | **Sub-question (if provided)** |
| --- | --- | --- | --- |
| 1 | Before engaging in this case study project, how much experience did you have with RE-AIM? | - Had never heard of RE-AIM - Had heard of RE-AIM but never used it - Had used RE-AIM on one project before - Had used RE-AIM in multiple projects | -- |
| 2 | Have you ever applied a different implementation science framework and/or project evaluation framework in your previous work? | - Never - Once - Multiple times | If you use a particular framework frequently, please name it |
| 3 | How difficult or easy was it to use RE-AIM in developing your case study? | - Very difficult - Difficult - Neither difficult nor easy - Easy - Very easy | -- |
| 4 | What was your greatest challenge in using RE-AIM? | (Open-ended) | -- |
| 5 | How useful was RE-AIM in developing your case study, for the following:   1. Understanding data availability 2. Planning for data collection 3. Understanding factors that led to the success and/or failure of the case 4. Drawing generalizable conclusions that extend beyond the case 5. Structuring the case study manuscript | - Not at all useful - Somewhat useful - Moderately useful - Very useful - Extremely useful | -- |
| 6 | Rank the RE-AIM constructs in terms of how conceptually challenging they were to understand/apply to your case study:   1. Reach 2. Effectiveness 3. Adoption 4. Implementation 5. Maintenance | Rank from 1 to 5; 1 is most challenging; 5 is least challenging | -- |
| 7 | Rank the RE-AIM constructs in terms of the difficulty of gathering related data:   1. Reach 2. Effectiveness 3. Adoption 4. Implementation 5. Maintenance | Rank from 1 to 5; 1 is most difficult; 5 is least difficult | -- |
| 8 | Are there any important factors related to the dissemination and/or implementation of your clean cooking program that were not captured by RE-AIM? | Yes/No | If yes, please describe |
| 9 | How likely would you be to use RE-AIM for a future project? | - Not at all likely - Somewhat likely - Moderately likely - Very likely - Extremely likely | -- |
| 10 | How likely would you be to recommend using RE-AIM to a colleague engaged in:   1. Prospective design of a clean cooking program roll-out 2. Midpoint program evaluation and adaptation 3. Retrospective program evaluation 4. Cross-country comparisons of clean cooking programs | - Not at all likely - Somewhat likely - Moderately likely - Very likely - Extremely likely | -- |
| 11 | Will your experience using RE-AIM in this case study project lead you to approach your work differently in the future? | Yes/No | If yes please describe |
| 12 | Would any additional resources (e.g. how-to guides, articles on theory, etc.) have been helpful for guidance on using RE-AIM? | Yes/No | If yes please describe |
| 13 | Any additional comments about your experience using RE-AIM for the case study project? | (Open-ended) | -- |
